# Studying social interactions through immersive virtual environment technology: virtues, pitfalls, and future challenges

**DOI:** 10.3389/fpsyg.2015.00869

**Published:** 2015-06-24

**Authors:** Dario Bombari, Marianne Schmid Mast, Elena Canadas, Manuel Bachmann

**Affiliations:** ^1^Department of Organizational Behavior, Faculty of Business and Economics, University of LausanneLausanne, Switzerland; ^2^Bern University of Applied SciencesBern, Switzerland

**Keywords:** social interaction, immersive virtual environment, virtual humans, avatars, copresence

## Abstract

The goal of the present review is to explain how immersive virtual environment technology (IVET) can be used for the study of social interactions and how the use of virtual humans in immersive virtual environments can advance research and application in many different fields. Researchers studying individual differences in social interactions are typically interested in keeping the behavior and the appearance of the interaction partner constant across participants. With IVET researchers have full control over the interaction partners, can standardize them while still keeping the simulation realistic. Virtual simulations are valid: growing evidence shows that indeed studies conducted with IVET can replicate some well-known findings of social psychology. Moreover, IVET allows researchers to subtly manipulate characteristics of the environment (e.g., visual cues to prime participants) or of the social partner (e.g., his/her race) to investigate their influences on participants’ behavior and cognition. Furthermore, manipulations that would be difficult or impossible in real life (e.g., changing participants’ height) can be easily obtained with IVET. Beside the advantages for theoretical research, we explore the most recent training and clinical applications of IVET, its integration with other technologies (e.g., social sensing) and future challenges for researchers (e.g., making the communication between virtual humans and participants smoother).

Humans spend between 32 and 75% of their waking time in social interactions (Mehl and Pennebaker, 2003). To understand how we behave in social interactions, how we draw conclusions about our social interaction partners, or how the outcome of the social interaction will shape us and our social relationships, we need to observe and study humans engaged in a wide variety of different social contexts. Given the frequency of its occurrence and the importance of social interactions for understanding humans and for bringing about change for individuals and society, the lack of research using direct behavioral observation is surprising (Baumeister et al., 2007). One reason for this gap is that if we focus on natural observation, we may have to wait long periods of time before a desired social situation occurs naturally with us being present to observe it. In an attempt to overcome these constraints, researchers typically use simulations, meaning that people are put in a specific social situation from which their behavior is observed and the interaction outcomes assessed. In the present review, we describe how such simulations can take place in an immersive virtual environment (IVE) with virtual humans as social interaction partners and we discuss the distinct advantages and challenges of this method.

In this article, we focus on social interactions with virtual humans in the IVEs and their use for research and training. While IVET has been around for several decades, the use of this technology for the social sciences is still relatively new (Fox et al., 2009) and particularly the aspect of including virtual humans as social interaction partners to simulate interpersonal encounters is still in its infancy. It is the latter aspect on which we will shed light by describing the state of the art in this domain, some of the main findings, and the existing challenges and future directions of this line of research. Our contribution is at the same time an update of the earlier review by Fox et al. and a focalization on the simulation of social interactions with virtual humans.

## The Need for Standardized Social Interaction Partners

For researchers studying how people behave in social interactions, one of the biggest challenges is that the behavior of one person is always, at least in part, a function of the behavior of his/her social interaction partner. If my social interaction partner smiles a lot, then I tend to respond in kind (Hatfield et al., 1992). Typically, social scientists studying interpersonal behavior are interested in investigating why one person behaves differently from another person – known as the study of individual differences. Such differences become hard to interpret if they are affected by what the social interaction partner does. There are different solutions to this problem of non-independence of the observational data in social interactions. One possibility is to include the interaction partner’s behavior as a control variable in the statistical analysis. This is not an optimal solution because the “contamination” of a person’s behavior by another person’s behavior occurs simultaneously through different channels (e.g., verbal and non-verbal) and the behavioral cues are often very subtle and hard to observe and measure. Moreover, it is unclear which out of the many different behaviors a person shows would have to be assessed in order to be able to control for.

The optimal solution is the standardization of the social interaction partner, meaning that the social interaction partner behaves exactly in the same way with each and every participant. With the standardization of the social interaction partner, differences in the behavior of a series of participants can be attributed entirely too actual differences among these people and not to anything their social interaction partner did.

One approach to standardization is the use of trained confederates. These are actors that are instructed and trained to maintain the same verbal and non-verbal reactions across participants and across conditions. Interacting with confederates (that the participants believe to be regular other participants) has high ecological validity because it is an interaction between two humans. However, in terms of standardization, it does not ensure that all behaviors are entirely controlled, especially if one considers non-verbal behavior (e.g., facial mimicry) that is much less under conscious control than, for instance, verbal behavior. Indeed, research (Congdon and Schober, 2002; Topal et al., 2008) shows that confederates still behave slightly differently depending on whom they are interacting with and this has an influence on participants’ behavior (see Kuhlen and Brennan, 2013 for a discussion on this topic).

Another experimental setting used to circumvent the issues associated with the inter-dependence of the behavior in a social interaction involves the use of vignettes. In vignette studies, participants are provided with a cover story or with cues (e.g., a picture) describing an interaction partner in a particular situation. Participants are asked to imagine being in an interaction with that partner. This setting has the advantage of maximally controlling the behavior of the social interaction partner (maximal standardization) to the detriment of ecological validity. These studies are quite far removed from real-life interactions and might thus find results that cannot be generalized to or might not be valid for real-life situations.

Typically, the methods high in ecological validity (e.g., social interactions with confederates) are low on standardization and the methods high in standardization (e.g., social interactions with a person described in a vignette) are low on ecological validity. Using virtual humans in an IVE provides us with the best of both worlds: high ecological validity and high standardization (Blascovich et al., 2002). Thus, IVEs presents a valuable possibility to overcome the issues we discussed above. In addition, using a virtual simulation of an interaction enables researchers to easily replicate the studies, which is important especially for those domains, such as social psychology, in which replication is lacking (Blascovich et al., 2002).

## Virtual Humans in IVEs

A virtual human is a computer-generated three-dimensional digital representation that looks and acts like a real human. Blascovich et al. (2002) differentiates between human-avatars (virtual humans controlled by humans) and agent-avatars (virtual humans controlled by computers). In the present article, we use the generic term virtual humans.

The first attempts of using virtual humans as social interaction partners became possible in the 90s. These technologies consisted of a desktop computer in which one or more virtual human interaction partners were displayed and could interact with the participant (e.g., provides information, answer standardized questions). Whereas this method constituted an improvement in terms of standardization, realism was still quite low and, as a consequence, the implications of any findings obtained were limited. This changed at the turn of the new millennium with the advancement of technology and the increased processing power of computers, making it possible to incorporate virtual humans in IVEs.

### Immersion in the Virtual

Immersive virtual environment technology means that a person is fully immersed in a virtual world in which he or she can walk and look around as in the real world. The basic setup of IVET is the following: (1) the physical movement (e.g., head turning) of a participant is tracked (e.g., via an infrared camera), (2) the perceptual information of the virtual world is updated according to those movements through computer-based calculations, and (3) the perceptual information (e.g., visual information displayed through head-mounted displays) is sent back to the participant (Blascovich et al., 2002). Even though in principle any kind of sensory feedback can be provided to participants, most of the studies on social interactions focused on visual and auditory information, which is typically sent through the head-mounted display (or projected to the physical walls of a room, as in so-called CAVE systems) and headphones or speakers.

We refer to immersion as the *objective* amount and quality of the perceptual input provided to participants through technological instruments (Mantovani and Castelnuovo, 2003), such as the 3D visual input. Also, the degree of immersion in the virtual world and in the interaction with virtual humans can be manipulated by providing more or less sensorial information to the participants. As an example, IVET is more immersive than desktop virtual reality because it provides more sensorial inputs. We use presence as it refers to the participants’ subjective feeling of “being there,” interacting with their own body in a virtual world that is perceived as real (Heeter, 1992; Ijsselsteijn et al., 2001; Schuemie et al., 2001). It can be operationalized as the correspondence of participants’ reactions and emotions between a real and a virtual situation and can be measured in different ways (e.g., physiological responses, behavioral measures, and self-assessment). The literature is quite inconsistent in terms of the different definitions of presence and immersion. Some authors refer to the former as “psychological immersion” (Palmer, 1995) and to the latter as “perceptual immersion” (Biocca and Delaney, 1995). Other authors define immersion as a subjective feeling (Fox et al., 2009), as the degree of “realness” of participants’ behavior (which, as explained above, we rather consider as an operationalization of presence), or use the terms presence and immersion interchangeably. In our view, immersion is a determinant of feeling of presence. In Freeman et al.’s (1999) study, participants watching motion scenes in 3D reported higher feelings of presence when compared to 2D. Kober et al. (2012) found that EEG activity in parietal areas of the brain was correlated with feelings of presence and was higher when participants were involved in a highly immersive virtual reality environment compared to a desktop version of the same task. Even though research has shown that virtual reality can evoke a strong feeling of presence, and especially so in immersive virtual environments, the intensity of those reactions are not as pronounced as in real world situations (Jacobson, 2001). Importantly, feeling of presence in IVEs can be improved by using virtual humans as social interaction partners (Slater et al., 2006b). Copresence is an aspect of presence that implies the feeling of being there, in the same virtual space, together with virtual humans. As a consequence, individuals feel that virtual partners are “available” and can either influence or be influenced by them (Lee, 2004). Social presence is a broader concept than copresence as it does not require sharing the same virtual space (Lee, 2004). As we will show in the next sections, the use of virtual humans in IVEs represents a powerful social interaction simulation method.

### Realistic Looking Virtual Humans

High ecological validity can also be achieved by using virtual humans that look realistic and behave in a realistic way. Technological advances have improved the graphic quality and the motion animation of virtual humans dramatically over the past decade. The virtual humans available to date are very convincing. Typically, the better the esthetic representation of a human and the closer to a real human the human representation comes, the more acceptable the human representation is to an observer, engendering more natural reactions from the observer (Blascovich, 2002; Slater and Steed, 2002). However, at a certain point of similarity, an observer’s reaction can be of revulsion, only to return to something more positive when the virtual human becomes more distinguishable from a real human. This is called the *uncanny valley* effect (Mori, 1970). With the increased realism in virtual humans we become less likely to accept features that deviate from actual human features. That is, unless the representation is absolutely “perfect,” we will pick up on subtle abnormalities in the representation which makes us respond in an adverse way. Indeed, participants have an unpleasant impression of highly realistic (although not perfect) virtual humans as opposed to more caricature-based avatars (Seyama and Nagayama, 2007). To illustrate, a brisk and unnatural hand movement in a very simplistic virtual human would be less surprising and can be attributed to the crudeness of the simulation of the virtual human. However, if an almost perfect virtual human shows the same gesture; observers are bothered and they try to find out what is wrong with the virtual human, which then reduces its perceived realism and participants’ copresence. Even though there are many anecdotal examples about the *uncanny valley*, the effect has not been systematically studied in an IVE. Overall, studies using IVET and other methodologies (e.g., videoclips, desktop virtual reality) show that virtual humans are reported as odd or eerie when there is a perceived mismatch between their high-quality “physical” appearance and their behavior, such as their gaze behavior (Garau et al., 2003) or their facial expressions (Tinwell et al., 2011).

## Are Virtual Social Interactions Similar to Real Social Interactions?

Despite the relatively high ecological validity of IVET-based social interactions, they still remain virtual. One might therefore wonder whether social interaction behavior shown with virtual humans in IVEs is similar to what people would do in real world interactions. Bailenson et al. (2003) measured the interpersonal distance that participants maintained while approaching a virtual human who engaged them in mutual gaze as compared to a virtual human who did not look at the participants. Results show the same behavioral pattern found in real social interactions (Argyle and Dean, 1965; Patterson et al., 2002): when the social interaction partner (the virtual human) looked at the participants, the latter maintained greater interpersonal distance than when the social interaction partner was not looking at them.

In the same vein, Hoyt et al. (2003) used IVET to replicate classic social psychology findings on social inhibition. They trained a group of participants in a specific task and subsequently asked them to perform it either in the presence of virtual humans or alone. In accordance with the classic social inhibition finding (Buck et al., 1992), participants performed worse when in the presence of virtual humans. Relatedly, the presence of a social interaction partner often increases arousal in real social interactions (Patterson, 1976) and the same was true in an IVE. Slater et al. (2006b) found that participants had higher arousal, measured through physiological responses such as heart-rate and galvanic skin response, when they were in a virtual environment with virtual humans present (i.e., a bar) compared to a lone training session in the IVE. Also, the closer the virtual human approached participants, the higher their physiological arousal (Llobera et al., 2010).

Giannopoulos et al. (2010) investigated handshakes by asking participants to take part in a virtual cocktail party. They had to shake virtual humans’ hands by using a haptic device controlled either by an algorithm created to produce realistic movements or by a real human. Results showed that virtual handshakes operated by a robot were rated similarly as handshakes operated by humans. Dyck et al. (2008) used the Facial Action Coding System (Ekman and Friesen, 1978) to artificially create facial expressions of six basic emotions on virtual humans that closely matched those displayed by real actors. Specific facial action units used in natural expressions were implemented in virtual humans. Results showed that virtual facial expressions of emotions displayed by virtual humans were overall recognized as accurately, and for some emotions (i.e., sadness and fear) even more accurately, as natural expressions displayed by real human actors. This study suggests that virtual humans can be reliably used to communicate emotions, although some technical advancement is needed to improve the perceived quality of some specific emotions (e.g., disgust). In the same vein, Qu et al. (2014) asked participants to have a conversation with a virtual woman who displayed either positive or negative facial expressions both while speaking and listening to the participants. Results showed that the emotions (positive or negative) displayed by the virtual woman during the interaction, and especially in the speaking phase, evoked a congruent emotional state in the participants. The same effect was observed in real social interactions (Hess and Blairy, 2001; Hess and Fischer, 2013). Santos-Ruiz et al. (2010) adapted the Trier Social Stress Test (TSST; Kirschbaum et al., 1993), a task typically used to induce acute social stress, to an IVE. As in the original version of the TSST, participants had to deliver a speech addressing their own good and bad qualities. The virtual human audience changed attitude from interested to restless. Following the speech participants performed an arithmetic task (to continuously subtract 13 starting from a given number) and were informed that after an error they would have to start over. Electrodermal responses and increased salivary cortisol levels in the participants were in line with those found in previous research outside IVEs (Kelly et al., 2007).

The engagement in the virtual situation and the extent to which participants perceive the virtual social interactions as real differ among individuals. Typically, the feeling of presence is measured in participants in order to check whether it affects the results obtained. This could be used to discard participants who were for one reason or another not engaged enough in the virtual world or did not have the feeling of being there, which, based on our decade long experience in virtual reality, has very rarely happened. For correlational research it is, however, important to assure that the findings are not due to the fact that some people felt more presence than others. Research shows that individual differences in feelings of presence typically do not affect the results. For instance, in a scenario in which participants were in the role of a patient (Schmid Mast et al., 2008), they behaved differently when interacting with a dominant vs. a non-dominant physician. Importantly, the degree to which they were engaged in the virtual encounter – their feeling of presence – did not affect the results. In the same vein, Hartanto et al. (2014) used IVET to induce social stress in participants through job interviews with two virtual humans. They reported that differences in presence among participants did not affect feelings of stress.

In summary, there is evidence that subjective feelings, behavioral, and physiological reactions during interactions with virtual humans are very similar to those shown during interactions with real humans. IVET-simulated interactions are therefore a dependable manipulation that can be considered a proxy of real life interactions. In the next section, we discuss some of the main advantages of using virtual humans and IVEs for studying social interactions.

## Why Use Virtual Humans in IVEs?

The standardization of the social interaction partner is useful for social psychology studies because all the observed variance among participants can fully be attributed to them, or to a previous manipulation, and is not due to or affected by the social interaction partner’s behavior. Interacting with virtual humans in IVEs has also three other distinct advantages. First, it enables the researcher to manipulate something in the environment or about the virtual social interaction partner and then to observe how this manipulation affects the participant’s interaction behavior and/or interaction outcomes. Second, IVEs provide a means of exposing the participant to social interactions that may well be impossible in real life. Third, virtual humans in IVEs are a relatively low-cost and effective solution to train participants or clinical populations in different tasks.

### Manipulation of the Virtual Environment and the Virtual Human

Using a standardized simulation of a social interaction with virtual humans and IVEs provide the opportunity to subtly manipulate something in the virtual environment or the virtual human to test the effect of this change on the social interaction. Creating such controlled conditions are crucial for the discovery of causal relationships among variables and for disentangling the single or joint effects of different aspects of the environment or the social interaction partner on the way a social interaction unfolds. To illustrate, Latu et al. (2013) asked participants to deliver a persuasive speech in front of a group of virtual humans. The experimental manipulation centered around a picture hanging on a wall of the virtual room facing the speaker. Female participants showed improved speech performance when the picture displayed a female role model (i.e., Hillary Clinton, Angela Merkel) compared to a male role model, or when no picture was presented. Importantly, the virtual humans maintained the same non-verbal behavior across all participants, which enabled the researchers to conclude that the obtained effect was based solely on the experimental manipulation.

Moreover, the reaction of the public itself can be manipulated in order to study the effect on participant’s behavior. Pertaub et al. (2002) involved participants in a public speaking situation in which they had to deliver a speech in front of a neutral, a positive, or a bored audience composed of eight virtual humans. Unsurprisingly, they found that the negative/bored audience provoked higher levels of anxiety in participants. Overall, in studies involving a public speaking situation, IVEs are a worthy option not only because of the experimental control they afford but also because recruiting a group of actual humans would be time and cost intensive.

Alternative manipulations to virtual scenarios could involve changes to the virtual humans so as to test whether this manipulation affects the participant’s behavior in a social interaction. The use of virtual humans in IVEs enables us to disentangle variables that, in real life, are often interwoven and to study their respective effect on an outcome variable. For example, female doctors typically have a more caring and empathic communication style when interacting with their patients than male doctors (Roter et al., 2002). If we want to test the effect of women doctors and of a caring and empathic communication style independent of each other, we have to be able to vary them independently. We did so in a study in which we had female and male virtual doctors use either a caring or non-caring combined with either a dominant or non-dominant communication style and measured the participants’ satisfaction with the (virtual) consultation (Schmid Mast et al., 2008). Results showed that female patients were particularly satisfied with female doctors who adopted a gender-congruent, thus caring communication style whereas patient satisfaction for female doctors was unaffected by the dominance dimension. Satisfaction with the male doctors was unaffected by either communication style.

In a social situation, we react to the other person’s verbal and non-verbal behavior and also to the other person’s appearance. The effect of these different pieces of information can also be varied independent of each other when virtual humans are used. The same virtual human can, for instance, provide the same spoken information to all participants but differ in the non-verbal information depending on the condition participants are in. For instance, there could be two versions of the virtual human, one that has an expansive and animated body posture and one that has a constricted and rather immobile posture, while holding the spoken information the virtual human delivers constant. In such a setting, researchers could investigate how body language, specifically, affects the social interaction partner. This manipulation would be extremely difficult to obtain when using trained confederates. Indeed, Bailenson and Yee (2005) used a similar paradigm to study the effect of body posture mimicry of virtual humans on participants’ ratings of verbal information and of the general impression made by the virtual humans. Virtual humans delivered a persuasive speech to participants while either mimicking the participant’s body position with a delay of 4 s or while performing prerecorded body movements. Participants rated mimicking virtual humans more positively and their speeches as more persuasive compared to non-mimicking virtual humans. Likewise, Vinayagamoorthy et al. (2008) found that the body posture position of a virtual human providing information to participants played an important role on the perception of affective states of the virtual human. Participants interacting with virtual humans displaying anger reported that their body posture was the primary source of information to detect their emotional state.

Moreover, while the verbal and non-verbal behavior is kept constant, researchers can manipulate the physical appearance of a virtual human in order to test its influence on participants’ behavior. In Dotsch and Wigboldus (2008)’s study, Caucasian participants approached virtual humans with either White or Moroccan facial features. Participants maintained a bigger interpersonal distance to Moroccan-like virtual humans and the effect was moderated by their implicit negative associations toward this group.

### Impossible Real-World Social Interactions in the Virtual

Another advantage of using IVET to study interactions is that situations and manipulations that would be impossible in real life can be created. Although ecological validity of such experiments are by definition low, they can help to understand how different variables interact with each other and advance our theoretical understanding of human cognition and behavior. To illustrate, participants can be embodied (i.e., own or control a virtual body from a first person perspective) in any virtual human with any specific characteristics and this can have an effect on interaction outcomes. The psychological and behavioral effects due to the embodiment of people in a particular virtual human are known as the Proteus effect (Yee and Bailenson, 2007). Yee and Bailenson (2007) made participants adopt more or less attractive virtual humans and found that participants assigned to attractive virtual humans approached more closely other virtual humans. In a second study, participants performed a negotiation task while embodying taller or shorter virtual humans. Participants assigned to taller avatars behaved in a more confident way during the interaction. The method researchers typically use to provide visual feedback about the physical appearance of the virtual human that participants embody is to locate a virtual mirror in the IVE (Yee and Bailenson, 2007). The virtual mirror reflects the real body movements of the participants while the appearance can be rendered in any form.

Many physical appearance manipulations of the virtual human are possible, including gender, race, age, and body size. Importantly, manipulating people’s appearance changes their cognitions, possibly by associating the self with concepts related to other groups (Maister et al., 2015). In this sense, virtual embodiment could be used as an alternative to priming manipulations. As an example, Peck et al. (2013) showed that embodying white participants into dark-skinned avatars reduced their implicit racial bias. Kilteni et al. (2013) found that participants embodied in a dark-skinned and casual-dressed virtual human improved their drumming skills. Given the rather explicit nature of embodiment, some caution should be used in order to avoid social desirability effects (e.g., participants might respond according to what they think it is expected from them).

Another example of manipulations that would be impossible to test in a real life situation is when extreme or complex social behaviors and cognitions are involved. For instance, Slater et al. (2006a) replicated the well-known study by Milgram (1963) in an IVE in which participants administer electric shocks to interaction partners. The results were comparable to the real world study, namely that participants tend to obey to orders from authority figures to the extent of administering severe electric shocks that could endanger another person’s life.

A collaborative virtual environment (CVE) is yet another example of how real world social scenarios can be incorporated into the virtual. In these settings the actual humans do not need to be in the same physical space but can remotely embody an avatar and interact with peers. This manipulation was used by Bailenson et al. (2005) in a study on augmented gaze in which three participants were present in the scenario. One of the participants read a persuasive message to the other two participants. Importantly, the gaze of the reader was manipulated in order to be perceived by the listeners as either natural or transformed. In the transformed condition, listeners perceived the reader as either looking always or never at them. When readers fixated the listeners, the latters rated their message as more persuasive and showed better recall of it. In Bente et al. (2007)’s study, dyads of participants were involved in interactions while being embodied in virtual humans. Interaction partners were shown with the real partner’s gaze behavior or with a manipulated gaze, displaying either longer or shorter eye contact. Participants showing manipulated longer direct gaze were evaluated more positively by their interaction partners. The advantages of CVEs are that feeling of presence and copresence are high (i.e., participants are involved in an interaction with a human partner) and that very specific behaviors can be rendered non-realistically (the so-called transformed social interactions) and thus the consequences of these individual manipulations can be investigated.

### Training with Virtual Humans in IVEs

Simulation of social interactions is not only important for research purposes but also for training. For instance, virtual humans can either function as tutors and give performance feedback or they can be used as specific social interaction partners necessary for training. For example, the virtual human can be a recruiter asking the participant job interview questions and the participant trains on giving good answers and making a favorable first impression. The great advantage of using virtual humans for training is that they are constantly available and do not need to be trained, scheduled, or paid. Bailenson et al. (2008, Study 1), for instance, trained participants in Tai Chi movements using a virtual teacher. Participants reported a more enjoyable learning experience when they had the possibility to see themselves performing next to their teacher performing the movements compared to a condition in which they could see only the teacher. This finding indicates that some features of the interaction, such as having the possibility to compare one’s own movements to those of the teacher, play a crucial role in the learning outcome.

Poeschl and Doering (2012) modeled a virtual audience from real audience data that can be used to provide feedback in fear of public speaking training. Batrinca et al. (2013) also developed an audience composed of virtual humans that can provide feedback online to presenters about their performance. The advantage of using virtual humans is especially important for trainings such as learning how to speak in front of large audiences. It is now possible to simply program a large audience populated with virtual humans without having to recruit many people to be stooges as audience (Harris et al., 2002; Pertaub et al., 2002; Thalmann, 2006). However, there are investment costs of setting up an IVE laboratory and the programming of the virtual humans and environments. The development of portable systems is a promising venue to make virtual reality more accessible to practitioners.

Immersive virtual environment technology-based training has already been used in clinical settings. Park et al. (2011) created an IVET version of the traditional social skills training based on role-playing. Schizophrenic patients assigned to the IVE condition improved their conversational skills and assertiveness more than patients in the traditional role-playing group, however, the latter was more effective in emotion expression skills. Perez-Marcos et al. (2012) proposed an approach of neurorehabilitation for patients with reduced mobility based on virtual interactions with healthcare providers who are not in the same physical space. Patients and healthcare providers communicate remotely through a multisensory IVE and through haptic devices located at both sites that enable them to interact (see, hear, and touch) as in a real consultation. Some of the proposed tasks are cooperative, meaning that the patients and the doctor need to perform an action together and simultaneously in order to achieve a goal (e.g., cooperate to lift a virtual object). This kind of task increases patients’ feelings of copresence. This system enables the doctors to evaluate patients with motor deficits (e.g., through force feedback) or with neuropathic pain in upper limbs. In addition, a person-to-person interaction with a real doctor, even though remote, could increase motivation of patients to pursue rehabilitation programs and could help patients who are often socially isolated because of their reduced mobility to meet other people (e.g., doctors, nurses, or other patients) in a virtual environment.

## Communication with Virtual Humans

One of the biggest challenges in using virtual humans as social interaction partners is to achieve natural communication (e.g., free speech conversation) between participants and virtual humans. In most of the studies to date, the communication from the virtual human to the participant needs to be mediated by the experimenter. So the experimenter listens to what the participant says and then decides when and what the virtual human should respond. Moreover, the virtual human can only respond with behaviors or statements that have been programmed beforehand. Thus, virtual humans’ responses might not be precisely adjusted to participants’ utterances or to the tone of the conversation. As a result, the prosody, the syntax, or the word choice might not sound natural, hampering the flow of the communication. Even though research in IVEs on this topic is scarce, researchers studying interactions with confederates tried to address this issue by adapting scripts to real life conversations. Brown-Schmidt (2012) analyzed and coded conversations between two people who had to collaborate to correctly arrange pieces in a visual game. Based on occurring frequency of different types of answers (e.g., acknowledgment, repetitions) obtained through this analysis, confederates were instructed to use specific answer forms in a subsequent experiment. Likewise, in a picture description task, Branigan et al. (2007) instructed confederates to replicate errors (e.g., use of inappropriate verbs) that were made by naïve speakers in a previous similar task. Similar procedures inspired by real life conversations could be used to make conversations between virtual and real humans more smooth. Even though these methods might improve perceived realism of the communication, they do not assure an optimal adaptation to participants’ utterances.

Another possibility to achieve natural communication is to use confederates to embody virtual humans (Bailenson et al., 2005). Confederates can control the body position of the avatar (non-verbal behavior of the avatar could be standardized to some extent) while communicating in a natural way with participants. This solution would improve communication realism but it is not optimal because vocal non-verbal behavior of confederates might change across participants and therefore influence them, the detrimental effects of which have already been highlighted above.

Part of the reasons why achieving a realistic communication with virtual humans is problematic is that participants can potentially address them with any kind of utterance. One possibility is to “script” the conversation and to provide the participant with prompts so that the conversation flows more naturally. As an example, Schmid Mast et al. (2008) investigated participants in the role of patients interacting with virtual doctors in a virtual medical consultation. Participants were briefed about their symptoms and there were 16 turns between the virtual doctor and the patient and for each turn, the patient had a prompt card instructing him/her what information to deliver to the virtual doctor (e.g., talk about your symptoms, for how long you have had them and how much they affect your daily life). This ensured a smooth flow of the conversation but it was unnatural because no spontaneous remarks or questions were allowed. Another approach was tested by Qu et al. (2013, Study 2). They used a priming procedure to induce participants to use specific keywords when addressing virtual humans. They exposed participants to videos and pictures hanging on a wall in a virtual room, in which a virtual human asked them four questions on different topics. For example, when the topic was France, a picture of the Arc de Triomphe in Paris hung on a wall behind the virtual human in the priming condition, whereas only distractor pictures were displayed in the control condition. Results show that participants named the content of the videos and pictures significantly more often compared to a condition in which their content was not related to the question asked by the avatar. This priming procedure is promising because it could be combined with automatic keywords recognition and therefore enable virtual humans to respond in appropriate ways to human participants. For instance, when a participant is primed to use a specific keyword and he/she indeed says it during a virtual interaction, this keyword is automatically recognized by the system and triggers a specific response or behavior by the virtual human.

## Automatic Extraction of Participant Interaction Behavior in IVEs

Participant interaction behavior in IVEs is sometimes the dependent variable because the behavioral observation is the goal. The use of IVET makes it possible to extract some interpersonal behavior data of participants directly from the simulation because the system uses that information to function. Another method to extract participant interaction behavior is to use social sensing technology, which will be outlined below.

### Participant Interaction Behavior Extracted from IVET

There are some participant behaviors that can be measured directly by the IVE system that renders the virtual world. Interpersonal distance is a prime example for such automatic extraction of participant interaction behavior in a virtual encounter. This is because the IVE system constantly detects and monitors the location of the participant in order to render the virtual world in real time. Based on the location information of the participant and the virtual human, which is usually pre-defined by the programmer, interpersonal distance can be computed and registered during the entire social interaction. Interpersonal distance is an important social interaction behavior that can be indicative of approach-avoidance behavior or dominance (Hall et al., 2005).

Another variable that can be recorded by IVET is the actual scene that is visualized by the participants, which might be an indicator of attentional strategies. This measure can be recorded by placing either visible or invisible markers in specific locations of the virtual scene. Given that participants can still move their eyes to focus on specific portions of the visual scene even without moving their heads, visualized scene can be a proxy of gaze direction but does not represent a precise measure.

### Behavior Extraction Using Additional Equipment

In the previous section we discussed the use of visualized scene as a measure of attentional strategies within an IVE. The use of eye-tracking systems combined with the IVET allows more precise measures of attentional strategies. Wieser et al. (2010) involved a group of high and low socially anxious female participants in an IVE study in which they were approached by a virtual human. They measured participants’ eye movements and found that highly anxious participants avoided eye contact with male virtual humans.

Other measures, requiring additional equipment, include physiological data (e.g., heart rate, skin conductance response). Slater et al. (2006b) used an electrocardiogram to obtain measures of heart rate and recorded galvanic skin response while involving participants in a social interaction with five virtual humans. Results showed that the physiological measures changed significantly (i.e., faster heart rate and more pronounced skin conductance response) when virtual humans were present in the virtual world and when breaks in presence were elicited (i.e., short moments in time when participants’ subjective feeling of presence was interrupted by suddenly making the virtual world and the avatars vanish).

Given that this information about participant behavior is immediately available as the social interaction unfolds, these measures could be analyzed in real time and used to change or adapt subsequent behavior of a virtual human during an interaction. As an example, participant’s eye movements can be recorded and, for instance, the virtual human could then move to the location of the visual focus of the participant (or away from it, depending on the question under investigation). This data can also be complemented with information from social sensing to gather information about participant behavior.

Even though the devices outlined in this section are relatively non-invasive, the question remains whether their use interferes with participants’ feeling of presence. Indeed, one of the requirements for a virtual environment to be immersive is that information coming from the real world is shut out by a technological device (e.g., a head-mounted display) in order to enable individuals to focus on rendered information (Slater and Wilbur, 1997) and feel presence. For instance, knowing that eye movements are recorded or feeling an electrode on the skin could remind participants that the virtual simulation is fictitious and as a consequence feeling of presence might be reduced. Future research might experimentally investigate whether indeed feelings of presence are influenced by the use of the external devices (e.g., eye-tracking, electrodes) we outline in this section. Sensing via ubiquitous computing (where the there is no direct input from the participant to the sensing device; the sensing is unobtrusive) is by definition non-invasive and might play a more important role for IVET in the future. There are still technological advancements needed in order to make such devices (e.g., a heart rate monitor watch) as accurate as more invasive standard recording methods (e.g., electrodes for heart rate measurement). One emerging field that will play an important role for the study of social interactions in IVET is social sensing.

### Social Sensing of Participants in IVEs

Social sensing means the recording of interpersonal behavior from people engaged in social interactions via ubiquitous computing (i.e., no active computer input necessary, the environment is “smart” and registers people’s behavior) and computational models and algorithms for the automated extraction of social cues and for drawing social inferences (Schmid Mast et al., 2015). Unobtrusive social sensing devices are cameras, microphones, and Kinect sensors, among others. Behavioral extraction algorithms are available for different verbal and non-verbal behaviors (e.g., nodding, gesturing, speech time, loudness of voice, interruptions). We predict that social sensing will play an important role in the future development of automatizing the communication between the participant and the virtual human and for training purposes. As an example, imagine that the computer can detect the quality of the speech a participant is delivering in front of a large audience via social sensing. If the quality of the speech is bad, the program will put the virtual humans in the audience gradually to sleep. If the quality of the speech improves, the virtual humans in the audience will start to pay more attention and signal interest by following the participants with their eyes and erecting their posture. This is the goal of Cicero (Batrinca et al., 2013), a system that encompasses the automatic extraction of non-verbal behavior of a presenter through a Kinect device and gives a feedback (e.g., nodding, leaning forward) based on the evaluated (computed) performance (e.g., time spent gazing the audience, amount of pause fillers) through a virtual audience. Even though Cicero is not yet developed within IVET – only on a desktop virtual reality system - it is reasonable to assume that a similar system could be implemented in an IVE.

Another example in this direction comes from Zhang and Yap (2012) who studied automatic affect detection based on participants’ verbal (written) and non-verbal behavior during a virtual role-play. Affect detection in verbal information was performed through latent semantic analysis, which is an algorithm that automatically learns semantic information about words through their common use in natural language (Landauer and Dumais, 1997). Emotional gesture recognition was based on a Kinect device, which extracted emotional content based on a skeleton tracking procedure. To illustrate, a participant placing his/her hand on the head was identified as a signal of confusion.

Virtual humans that show a human-like behavior (i.e., agents that are able to produce sentences and respond to interaction partners in natural conversations) are called embodied conversational agents. Some research has stressed the importance of implementing complex behavior on embodied conversational agents, like multimodal (e.g., facial expressions and body gestures) emotional expressions (Pelachaud, 2009). Malatesta et al. (2009) developed a model to implement Scherer’s appraisal theory (Scherer, 2001) for the elicitation of emotions in embodied conversational agents by using different intensities and timings. In the future, it could be possible to implement subtle facial mimicry responses on virtual humans and study their effect on participants’ behavior.

## Conclusions and Future Challenges

As we illustrate in the present article, research on social interaction using IVET has established important results that were hard to achieve before its development. The here presented research is different from the one by Fox et al. (2009) in that we focus on social interactions with virtual humans in IVET whereas the Fox et al. (2009) paper is a broader review of the how IVET can and is used in the social sciences. Moreover, we are faced with a very fast developing research domain because of the frequent technical improvements and increased availability of relatively cheap virtual reality devices which makes an update since 2009 timely. In particular, in the last years more effort has been put into integrating IVET with other technologies, such as eye-tracking (Wieser et al., 2010), movement extraction devices (Zhang and Yap, 2012; Batrinca et al., 2013), and EEG (Kober et al., 2012). Moreover, recent studies have started to address the issue of making the conversation between participants and virtual humans smoother (Malatesta et al., 2009; Zhang and Yap, 2012). In addition, more studies investigated influences on participants’ behavior, physiological responses, and cognitions either by manipulating objects in the virtual world (Latu et al., 2013; Qu et al., 2013), avatars’ behavior (Llobera et al., 2010), or participants’ physical appearance in the virtual world (Peck et al., 2013). Last but not least, new applications have been created for clinical use (Park et al., 2011; Perez-Marcos et al., 2012) and for training participants, for instance when delivering a speech (Batrinca et al., 2013).

Even though research using IVET in social interactions has achieved important results, we argue that researchers will need to face some challenges in the next years. There is evidence showing that participants’ psychological and physiological reactions in IVEs are similar to those in the real world (Bailenson et al., 2003; Slater et al., 2006b). However, people may still react somehow differently with virtual humans compared to real humans. To illustrate, while more simple or automatic behavior (e.g., avoiding a virtual human that is invading a participant’s personal space) might be comparable between real life and IVEs, more subtle or complex behavior (e.g., being kind or appreciative to an interaction partner) could differ. Different solutions might be adopted in order to address this issue. One possibility is to improve verbal and non-verbal behavioral realism of virtual humans. As discussed above, motion quality should be adapted and match pictorial quality of virtual humans in order to avoid participant’s perception of eeriness due to the uncanny valley effect (Garau et al., 2003; Tinwell et al., 2011). Non-verbal behavior and motion of virtual humans could be rendered more realistically and more subtly by extracting it from real human motion. The latest blockbuster movies using computer-generated imagery (e.g., Avatar or The Lord of the Rings) might be taken as inspiration for this improvement. Computer-science advances are needed in order to implement very subtle non-verbal behavior (e.g., facial mimicry) on virtual humans and to improve the synchronization and the coordination between verbal and non-verbal behavior. For instance, lips movements should be adapted precisely to the phonic pattern of a verbal message.

In the same vein, while some effort has been made to improve communication between participants and virtual humans, it remains an important challenge for future research. Being able to have a free speech on any topic with a virtual human is the ultimate goal of this research area. Automatic language recognition, affect detection, social sensing, and speech production algorithms should be coordinated in order to achieve this goal.

Last but not least, perceived realism of virtual humans could be improved by implementing more high-level human qualities, such as personality traits, emotions, and theory of mind. Research shows that we form first impressions about strangers from verbal, non-verbal, and appearance cues (Funder and Colvin, 1988). Thus, virtual humans’ verbal behavior, non-verbal behavior, and physical aspect could convey distinctive and congruent information about their personality. An example of this would be an extraverted virtual human with an open body posture who talks a lot and wears a casual dress. This would be an interesting feature not only in order to achieve interaction realism, but also because participant’s behavior in relation to different personality traits could be studied with high experimental control. Furthermore, simulating emotions in virtual humans would be important to make participants experience that their behavior or anything happening in the virtual world can have an impact, either positive or negative, on virtual humans. Finally, simulating in the virtual humans the ability to infer the internal states of others (the so-called Theory of Mind) would increase participants’ feeling that virtual humans can “understand” them. Taken together, the proposed features would improve perceived realism of the interaction and participants’ feeling of copresence.

## Conflict of Interest Statement

The authors declare that the research was conducted in the absence of any commercial or financial relationships that could be construed as a potential conflict of interest.
